# CRISPR/Cas9 mediated knock-out of VPREB1 gene induces a cytotoxic effect in myeloma cells

**DOI:** 10.1371/journal.pone.0245349

**Published:** 2021-01-08

**Authors:** Mai Khaled, Amr S. Moustafa, Nashwa El-Khazragy, Maha Imam Ahmed, Marwa Ali Abd Elkhalek, Eman M. El_Salahy

**Affiliations:** 1 Department of Medical Biochemistry and Molecular Biology, Faculty of Medicine, Ain Shams University, Cairo, Egypt; 2 Clinical Pathology-Hematology & AinShams Medical Research Institute (MASRI), Faculty of Medicine, Ain Shams University, Cairo, Egypt; West China Hospital, Sichuan University, CHINA

## Abstract

**Background:**

Multiple Myeloma (MM) is a heterogeneous, hematological neoplasm that accounts 2% of all cancers. Although, autologous stem cell transplantation and chemotherapy are currently the most effective therapy, it carries a notable hazards, in addition for being non curative. Recently, the Clustered Regular Interspaced Short Palindromic Repeats (CRISPR-cas9) has been successfully tried at the experimental level, for the treatment of several hematological malignancies.

**Objectives:**

We aimed to investigate the *in-vitro* effect of CRISPR-cas9-mediated knock-out of V-set pre B-cell surrogate light chain 1”*VPREB1*” gene on the malignant proliferation of primary cultured myeloma cells.

**Methods:**

Bioinformatics’ analysis was performed to explore the gene expression profile of MM, and the *VPREB1* gene was selected as a target gene for this study. We knocked-out the *VPREB1* gene in primary cultured myeloma cells using CRISPR-cas9, the *VPREB1* gene editing efficacy was verified by determining *VPREB1* gene expression at both the mRNA and protein levels by qPCR and immunofluorescence, respectively. Furthermore, the cytotoxic effect on primary myeloma cells proliferation was evaluated using cytotoxicity assay.

**Results:**

There was a statistically significant reduction of both *VPREB1* mRNA and protein expression levels (p<0.01). knock-out of *VPREB1* gene in myeloma cell line resulted in a statistically significant reduction of myeloma cell proliferation.

**Conclusion:**

CRISPR-cas9-mediated knock-out of *VPREB1* gene is effective for inhibiting the proliferation of primary myeloma cells. This would provide a basis for a promising therapeutic strategy for patients with multiple myeloma.

## Introduction

In the past decade, the treatment of MM has been changed due to development of new therapeutic strategies which could be used either in frontline or relapse stages [1]. Currently, six different agents, namely alkylators, steroids, proteasome inhibitors, immunomodulatory agents, histone deacetylase inhibitors, and monoclonal antibodies are used in different therapeutic protocols either doublet, triplet or can be combined to autologous stem cell transplantation (ASCT) [2]. In spite of the availability of different therapeutic regimens, patients showed a heterogonous response with some cases demonstrating relapse. A better survival outcome was observed in patients who undergo hematopoietic stem cell transplantation than those who received chemotherapeutic agents [3]. The development of new therapeutic approaches for patients with MM is strongly required to improve the treatment outcome. Gene editing is recently tried at the experimental level for treatment of malignant diseases including hematological malignancies [4].

The Clustered Regular Interspaced Short Palindromic Repeats (CRISPR-cas9) is an adaptive immune system in bacteria and related organisms. CRISPR-cas9 consists of programmed single-stranded guide RNA “sgRNA” and a Cas9 endonuclease that generates double-strands DNA breaks (DSB) at a sequence-specific site [5]. The genome modification has been made by different approaches such as: insertion or deletions of small sequences “indels” that was mediated through non-homologous end joining (NHEJ) or homology directed repair (HDR) pathways [5–7]. At 2016, the first clinical trial using CRISPR-cas9-mediated gene editing was launched in China. The programmed cell death protein-1 (PD-1) gene knockout engineered T cells was evaluated for the management of metastatic non-small cell lung cancer [8–10].

CRISPR/Cas9 has been tested as potential therapy in multiple hematological diseases, including editing the β-globin (HBB) gene mutations in β-thalassemia [11] and efficient correction of Glu6Val mutation in sickle-cell disease [12, 13]. Furthermore, this technology was applied for treatment of Fanconi anemia through editing a point mutation in patient’s derived fibroblasts [14] and bleeding disorders such as neonatal autoimmune thrombocytopenia and post-transfusion purpura [15], hemophilia [16], and von-Willebrand disease [17].

The V-set pre B-cell surrogate light chain 1”*VPREB1*”, also named as CD179a, protein belongs to the immunoglobulin (Ig) superfamily and has a molecular weight of 16–18 KDa that consists of 126 amino acids. It is expressed on the surface of early pre-B cells, namely proB and early preB cells [18]. The protein is encoded by *VPREB1* gene that is located on *chr22*:*22*. This gene encodes the iota polypeptide chain that is associated with the Ig-mu chain to form a molecular complex on the surface of pre-B cells [19]. The *VPREB1*/Ig-mu chain complex regulates Ig gene rearrangements in the early steps of B-cell differentiation [20].

The structure of the CD179A includes an IgV domain-like structure that lacks the beta (beta 7) of the normal V domain, but has carboxyl terminals that do not show any sequential continuity with other proteins [20]. The CD179b is combined with the "Lambda 5" which carries the IgC domain-like structure, called an easy light chain-like structure called an alternative light chain or pseudo light chain [21]. In this complex, the incomplete V-domain of CD179A is complemented by the additional beta7 strand of CD179B. At the level of early B cells, the CD179A / CD179B light replacement chain is disulfide, which is attached to the CD79A / CD79B signal transduction heterodimer from the membrane-bound Ig Mu heavy chain. This type is called pre-B cell receptor (pre-BCR) [22]. The pre-BCR IG-M acts as a checkpoint in the early development of B cells to monitor the production of the heavy chain and combines the Ig-M heavy chain capacity with the Ig light chain [18]. This function is triggered by signals: B cell proliferation, differentiation of B before pro-B, promoting the restoration of IG light chain genes, and the release of elk in the IG heavy chain. Deficiency of pre-B cell receptors, such as CD179a or C-179b and Ig-Mu heavy chain, has caused severe impairment in human development, maturity, differentiation and B-cell agammaglobulinemia [23]. We aimed to knock-out the human *VPREB1* gene in primary myeloma cell line using CRISPR/Cas9 gene editing technology. To the best of our knowledge, this is the first in-vitro experimental study that describes the CRISPR/Cas9 mediated editing of the *VPREB1* gene in primary cultured myeloma cells. The application of this approach would provide a promising novel therapeutic target for MM patients.

## Materials and methods

### A. Bioinformatics analysis

In the present study, a biological bioinformatics approach was applied in order to analyze the gene expression profiles in MM patients [DisGeNET “http://www.disgenet.org/search”, Human Gene Mutation Database “http://www.hgmd.cf.ac.uk/ac/index.php”, and the Gene Expression Omnibus database “http://amp.pharm.mssm.edu/Harmonizome/resource/Gene+Expression+Omnibus”]. A functional analysis of differentially-expressed genes (DEGs) was performed between MM patients and healthy control group. In addition, in order to analyze the DEGs at functional level, we performed a [gene ontology (GO) “https://www.uniprot.org/help/gene_ontology”, Kyoto Encyclopedia of Genes and Genomes (KEGG) “https://www.genome.jp/kegg” and REACTOME pathway enriched analyses” https://reactome.org”] using the Database for Annotation, Visualization and Integrated Discovery (DAVID) online tool https://david.ncifcrf.gov/. We identified the target gene for this study to be the “V-set pre B-cell surrogate light chain 1”*VPREB1*” gene. More details about the conducted bioinformatics analysis regarding gene expression pattern and its relation to pathogenesis of MM are provided in S1 File

### B. Sample collection and preparation

All the requirements of the Declaration of Helsinki for research on human subjects were fulfilled and the approval of Ain Shams University Ethical Committee was granted with an authorization number: FWA 000017585. Accordingly; a written informed consent was signed from each participant. Five bone marrow (BM) samples were collected from multiple myeloma patients at Ain Shams University hospitals, Cairo, Egypt. The -diagnosis of MM was confirmed according to the International *Myeloma* Working Group (IMWG) guidelines updated at (2018) [24]. Three samples out of five were selected and pooled. The selection was based on detection of > 5% myeloma cells in BM samples. Then, we isolated the *VPREB1* “CD179a” positive cells from freshly pooled BM sample. The isolation procedure was done using magnetic microbeads cell isolation technology (*MACS*) (*Miltanie*, *Biotech*) according to the manufacturer’s instruction. Briefly, two sequential steps were performed. Firstly, a primary isolates of CD19 positive cells was separated using human CD19 Microbeads (CD19, human #130-050-301). Secondly, the negative cells were further used for separation of CD179a positive cells using (*VpreB*)-PE, human (clone: HSL96, cat no: 130-110-136) microbeads and *Anti-PE MicroBeads UltraPure*, (cat no:130-105-639).

### C. Culture of primary myeloma cells

Isolated CD179a positive cells were cultured using a double layer agar technique for growing of myeloma colonies forming units (*MY-CFU*_*c*_) from human bone marrow aspirates [25]. Cells were over-layered in HL60-conditioned medium (HL60-CM) and incubated at 37°C in an atmosphere of 5% CO_2._ The cell growth was observed for three weeks. Colonies (> 50 cells) was observed at two weeks and examined by inverted microscope. The cells have two different sizes, large cells represent plasmacytoid cells and the smaller are lymphoid phenotype.

### D. Gene editing in primary myeloma cells

The CD179a gene editing was performed on cultured CD179a positive cells using CRISPR-cas9 gene editing technology. A guide RNA of the *CRISPR/Cas9 nuclease* was constructed of **crRNA/tracr RNA duplex** using the True-Guide Synthetic gRNAs (cat no. A35509) for *VPREB1* gene (***Thermo Fischer Scientific***) and the tracrRNA (TrueGuide) cat no: A35506 (***thermo fischer scientific***), the target DNA primer sequence is “TCGGTGTGTACACGGTCTAC”. Two editing approaches were employed, direct and vector-mediated. For the second approach, the ssDNA was cloned into a *pGCS* plasmid vector using the **GeneArt™ Genomic Cleavage Selection Kit (Cat no: A27663 Thermo Fischer Scientific,** USA). The cloning procedure was conducted according to the manufacturer’s protocol. The resulted plasmid was transformed into *One Shot® TOP10 chemically competent E*. *coli*. Finally, cultured myeloma cells were transfected with CRISPR/Cas9 plasmid using a Lipofectamine CRISPRMAX Cas9 (***Thermo Fisher Scientific*, *USA***) and harvested 72 hours later. To discriminate between on-targets site from off-targets sites, DNA was isolated from CD179a edited myeloma cells using QIAQuick PCR purification kit (Qiagen, Germany), the specific locus cleavage site on DNA was generated by PCR amplification with specific primer sequences that covers the CRISPR/Cas9 cut site. Then, the nuclease assay was carried out to detect and validate the CRISPR/Cas9 specificity [26]. Nuclease assay was conducted by GeneArt Genomic Cleavage Detection Kit (***Thermo Fisher Scientific*, *USA***) as per the manufacturer’s guidelines and CRISPR/Cas9 cut specificity was checked by agarose gel electrophoresis (S1 Fig).

### E. Verification of *VPREB1* gene editing in myeloma cells

**E.1.** The *VPREB1* gene expression was measured by quantitative real time PCR (qPCR) using SYBR-Green fluorescent-based primer assay [*Hs_VPREB1_1_SG* QuantiTect Primer Assay, cat no: 249900, ID: QT00214466], (Qiagen; Germany).

**E.2.** Detection of target protein by immunofluorescence technique using rabbit anti-human VPREB1 monoclonal antibody (***Thermo Fisher Scientific*, *USA)*.**

### F. Assessment of *VPREB1* gene editing by CRISPR/Cas9 on the growth and viability of primary cultured myeloma cells

**F.1.** The total cell count was estimated in edited myeloma cells by Trypan blue staining using hemocytometer.

**F.2.** The cell viability was assessed by MTT CellTiter 96 assay (Promega, Germany), according to the manufacturer’s instructions.

## Results

Two CRISPR/Cas9 mediated approaches for the knock-out of *VPREB1* gene in primary human myeloma cells were used in this study. The two approaches were direct and *pGCS* vector-mediated. Seventy two hours post transfection, cultured cells were harvested and examined.

### Verification of the knock out efficiency of *VPREB1* gene in myeloma cells: Evaluation of *VPREB1* mRNA expression in human myeloma cell line by qPCR

**Fig 1:**
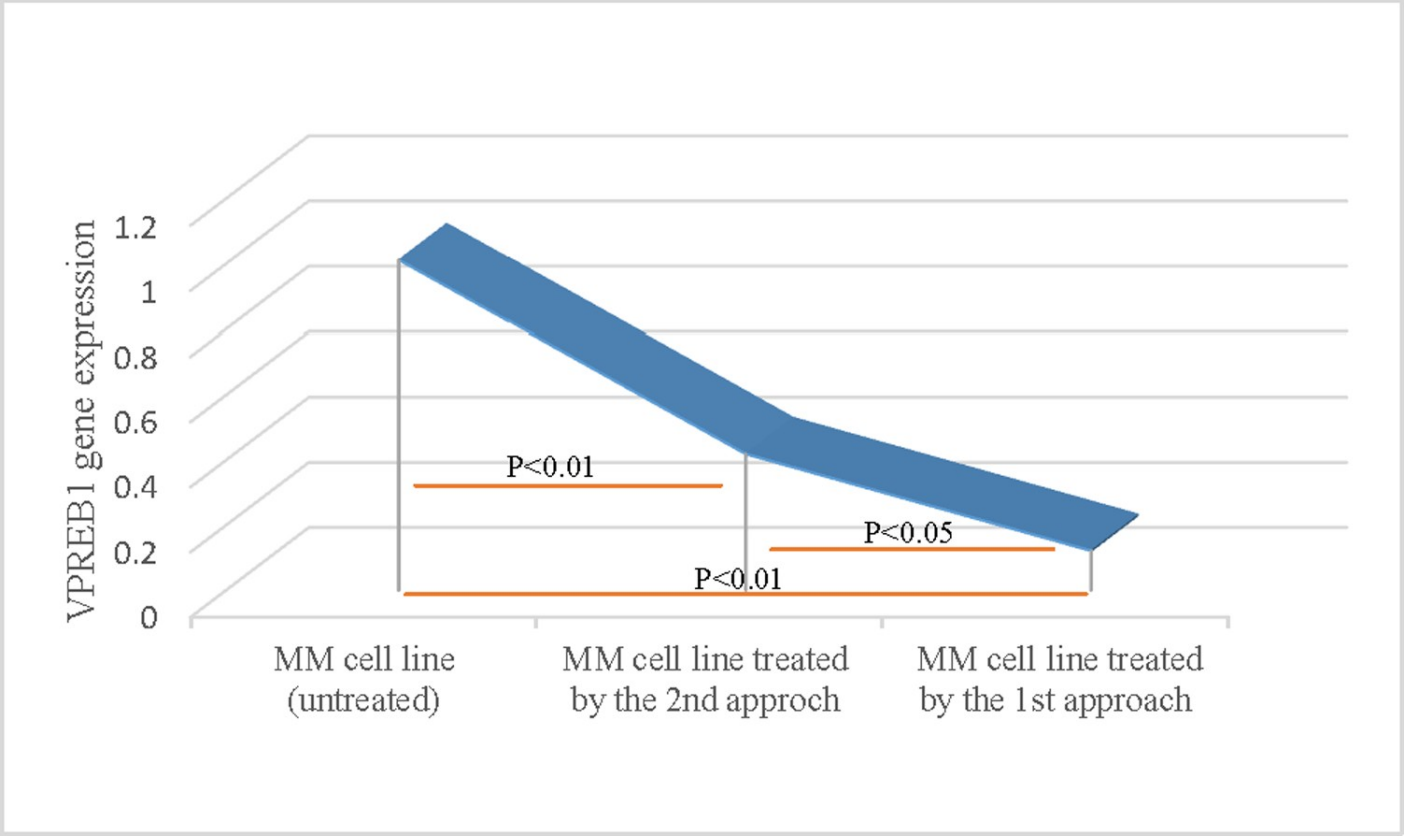
Verification of the knock out efficiency of *VPREB1* gene in myeloma cells by qPCR. Evaluation of *VPREB1* mRNA expression in human myeloma cells was determined by qPCR using SYBR-Green fluorescent-based primer assay [Hs_VPREB1_1_SG QuantiTect Primer Assay, cat no: 249900, ID: QT00214466], (Qiagen; Germany). A highly significant decrease (p<0.01) in *VPREB1* gene expression was demonstrated in CRISPR-treated cells as compared to untreated myeloma cells. There was also a significant decrease (p<0.05) in *VPREB1* gene expression level using the direct transfection (the 1^st^ approach), as compared to the vector-mediated (the 2^nd^ approach).

The *VPREB1* gene expression (Fig 1) was decreased in edited myeloma cells as compared to un-edited cells (p<0.01), indicating that transfection and editing were successful. The *VPREB1* gene expression level was significantly lower in cells edited by approach 1 as compared to approach 2 (p<0.05), The mean expression level of *VPREB1* gene was 1.012, 0.12 and 0.419 in un-edited cells, cells edited by 1^st^ approach and 2^nd^ approach; respectively.

### Verification of the knock out efficiency of *VPREB1* gene in myeloma cells: Evaluation of VPREB1 protein expression by immunofluorescence

**Fig 2:**
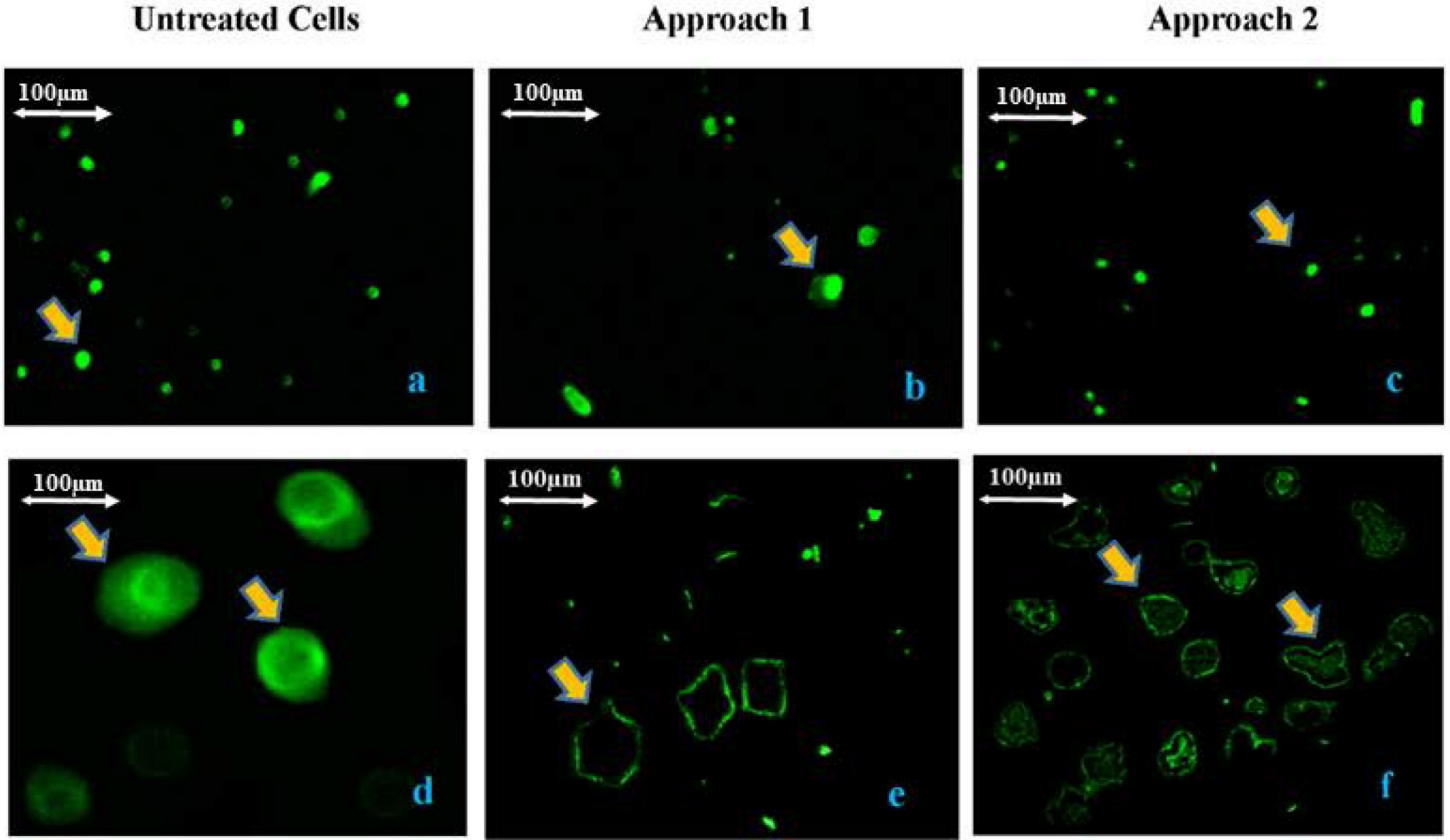
Verification of the knock out efficiency of *VPREB1* gene in myeloma cells by immunofluorescence. Evaluation of VPREB1 protein expression in treated and untreated myeloma cells by immunofluorescence using VPREB1 monoclonal antibody as the primary antibody and the anti-rabbit IgG Alexa Fluor 488 F(ab)’2 fragments as the secondary antibody (Thermo Fisher Scientific, USA). There was a significant decrease (p<0.01) in VPREB1 protein expression in treated myeloma cells (the 1^st^ transfection approach “b & e” and the 2^nd^ transfection approach “c & f”) as compared to the untreated cells (a & d). There was no significant difference (p>0.05) in VPREB1 protein expression between the 1^st^ and the 2^nd^ transfection approaches. The (a, b and c) are 100X magnification and the (d, e and f) are 400X magnification. The scale bar of the image is 100μm.

An immunofluorescent staining was performed on cultured myeloma cells to detect the VPREB1 protein expression in edited vs un-edited cells. The results (Fig 2) showed that the expression of *VPREB1* protein was significantly lower in *VPREB1*knocked-out cells than in un-edited cells (p<0.01). Although, lower expression level of the *VPREB1* protein was observed in myeloma cells that were edited by the 1^st^ approach as compared to that of 2^nd^ approach, this was statistically insignificant (p>0.05).

### The effect of CRISPR-mediated knock-out of *VPREB1* gene on the count of human myeloma cells

Our results revealed that *VPREB1* edited myeloma cells showed lower cell count compared to the untreated myeloma cells. The mean cell count of mock cells in the 1^st^ approach-treated cells and the 2^nd^ approach-treated cells were (7.3E+06), (3.2E+05), and (4.8E+05); respectively. A significant difference was detected in total cell count between the two edited approaches as well as between edited and un-edited cells.

### The effect of CRISPR-mediated knock-out of *VPREB1* gene mediates on human myeloma cell line: Cell viability and cytotoxic effect

**Fig 3:**
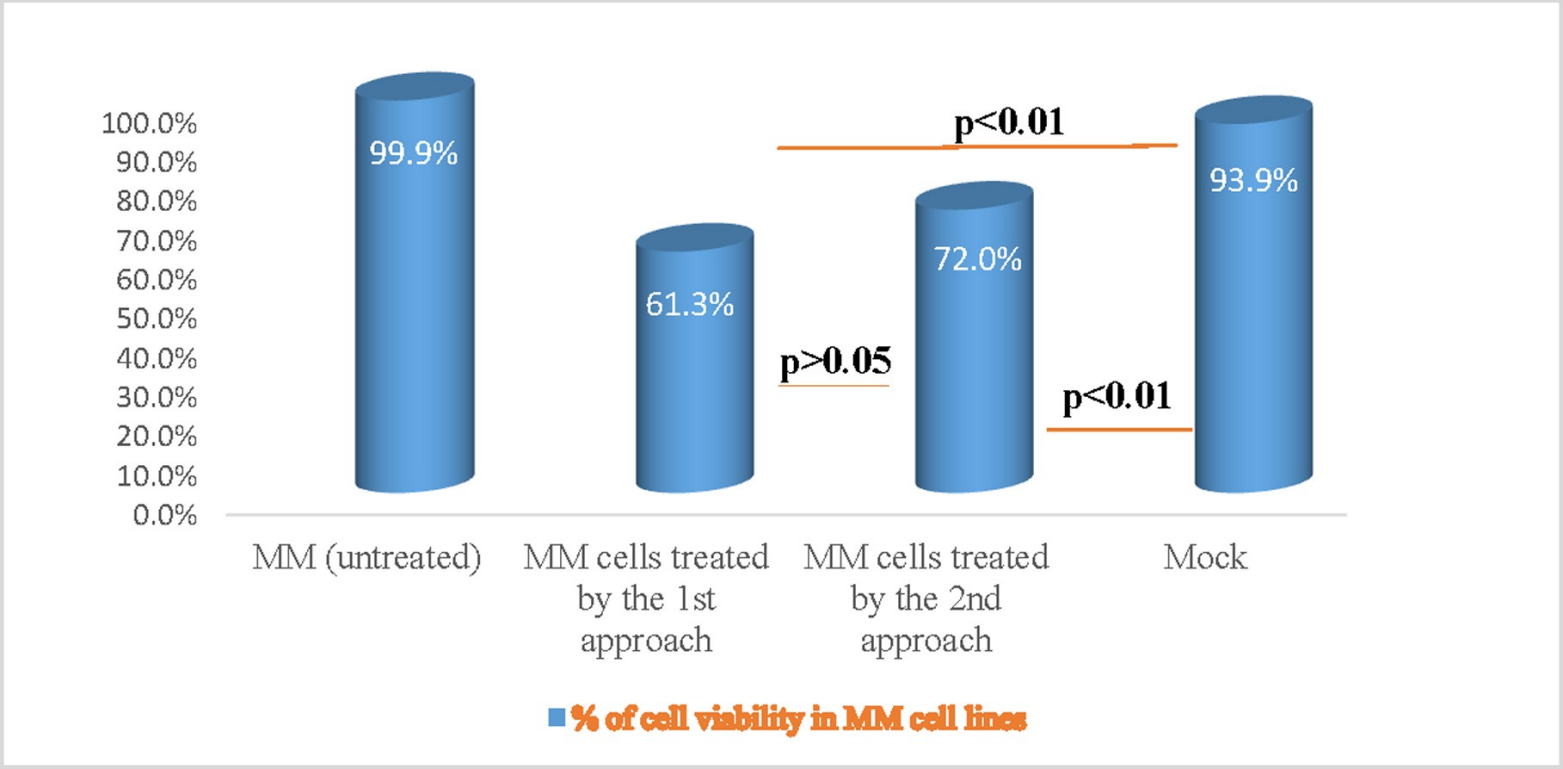
The effect of CRISPR-mediated knock out of *VPREB1* gene on human myeloma cell viability. Myeloma cell viability was assessed for treated as compared to untreated cells by MTT CellTiter 96 assay (*Promega*, *Germany*). There was a high significant decrease (p<0.01) in the percentage of cell proliferation inhibition that was observed by both the 1^st^ and the 2^nd^ transfection approaches, as compared to the untreated myeloma cells.

To investigate the impact of *VPREB1* gene editing on myeloma cell line, the *4*,*5-dimethylthiazol -2-yl)-2*,*5-diphenyltetrazolium bromide* (MTT) assay was conducted on treated and untreated cells in order to assess the effect of inhibition on cell proliferation and growth. The percent of cell proliferation inhibition was calculated based on the proliferation of untreated cells. The *VPREB1* gene knock-out by both approaches were more powerful to suppress cell proliferation and impair viability when compared to the untreated myeloma cells. The observed percentage of cell viability in the 1^st^ and 2^nd^ approaches—are 61.3% and 72.0%; respectively with a statistical significant difference (p<0.01) as compared to the untreated cells, however, no significant difference was observed in the cell viability between the two edited approaches (p>0.05) (Fig 3).

## Discussion

In the last decade, a major advance in genome editing has been observed, particularly with the development of engineered nucleases [27]. The CRISPR-cas9 system is the most novel, easy to handle, and precise tool among genome editing nucleases. It can efficiently edit genomes in different cell types regardless their complexity [28], and therefore, it has a broad application in therapeutic medicine [7, 9]. Although, MM is uncommon cancer, the disease heterogeneity and high mortality rate have forced the researchers to look for an effective, better tolerated therapy [29].

Based on bioinformatics analysis, we identified that *VPREB1* protein is overexpressed in multiple myeloma as well as other B cell malignancies. Moreover, we illustrated the vital role of *VPREB1* gene in differentiation and maturation at early stages of B cell development. Accordingly, we hypothesized that if we are able to *in-vitro* knock-out the *VPREB1* gene in myeloma cells using specific guide RNA sequence linked to CRISPR/Cas9 nuclease, the *VPREB1* protein expression is reduced and therefore we can inhibit the proliferation of human myeloma cells.

Based on our results, knock-out of *VPREB1* gene showed significant reduction at both *VPREB1* mRNA and protein expression levels in treated human myeloma cells, verifying the efficiency of knock out procedure. In addition, *VPREB1* gene editing had a proliferation inhibition effect on myeloma cells. This cytotoxic effect was more prominent with the direct transfection approach as compared to the vector-based approach.

Recently, CRISPR/Cas9 gene editing technology becomes a milestone in medical research. Several experimental models had been tested in malignant and inherited hematological diseases [5, 30]. For example, the implication of *in vivo* CRISPR/Cas9 gene editing in myeloid malignancies, including acute myeloid leukemia [30] and myelodysplastic syndrome [31] has been investigated. Gundry *et al* 2017 constructed an ideal, customizable, transplantable and pharmacologically tested model that targets the hematopoietic stem cells *in vivo* to introduce insertions and deletions in multiple alleles using CRISPR/Cas9 system [30].

In addition, Liu *et al* [32] investigated the therapeutic potential of CRISPR/Cas9 in HIV. They constructed a novel guided RNA to enhance the efficiency of a co-receptor of HIV (CCR5) knock out in peripheral blood. The study demonstrated a homozygous bi-allelic editing with 40–50% efficiency. In addition, the study conducted a comparative analysis between the two methods of gene editing which includes: CRISPR/Cas9 “an RNA-guided endonuclease” and transcription activator-like effector nuclease “*TALEN*, a *DNA-binding*, *motif-based endonuclease*”. The study demonstrated a higher overall efficiency (50–60%) of CRISPR/Cas9 in gene editing compared to *TALEN* with no off target events [32].

Considering the pathophysiology of plasma cell neoplasms, it has been evident that during differentiation and maturation of B cells, early B cell precursors express TdT, CD34 and HLA-DR. The heavy chain (H) undergoes rearrangements followed by addition of CD19 and CD10, then the IgM heavy and light chains are added and finally a surface immunoglobulin is added in addition to CD21 and CD22. At this moment; the B cell become mature [23, 33]. Interaction between Ig variable regions of B cells with a foreign antigen resulted in development of plasma cells. It has been demonstrated that the B cells precursor expresses CD179a and CD179b “precursors of light chains” which are an immunoglobulin related components “pseudo-immunoglobulins”. They represents a part of pre-B cell receptor which further replaced with conventional light chains [23]. Based on the above mentioned facts, it could be reasonable to conclude that the persistence of CD179a during B cell differentiation may result in freezing of B cells in its premature stage with blockage of maturation and increase clonal proliferation of B cells. When CD179a was knocked out using CRISPR/Cas9, the expression of CD179a gene is decreased together with the level of its protein expression which finally resulted in decrease myeloma cell proliferation and viability. Moreover, researchers had highlighted on the diagnostic and prognostic value of CD179a in precursor B acute lymphoblastic leukemia [34, 35]. They demonstrated that CD179a was exclusively expressed in precursor B-cell lymphoblastic lymphoma (B-ALL), but it is not expressed in mature B cell lymphomas. On the contrary, other pediatric tumors such as: precursor T-cell lymphoblastic lymphoma, extramedullary myeloid tumors and Ewing sarcoma are negative for both CD179a and CD179b [34]. In addition, the prognostic values of CD179a protein expression and copy number variation patterns of the pre-BCR components were investigated in pediatric precursor B-ALL. The study demonstrated that high expression levels of *VPREB1* gene was associated with arrest of B cell at pre-B stage and correlate with good prognosis irrespective to ALL subtype. Therefore, CD179a could serve as prognostic marker for high risk pediatric B-ALL patients [35].

In the current study, an efficient gene knock-out was achieved with direct delivery of CRISPR/cas9-gRNA complex into the cells compared to indirect delivery via cloned *pGCS* plasmid vector. The exact reason for this difference between the two approaches may be contributed to the efficacy of CRISPR-cas9 mediated knock out including the delivery method. In consistent to our results, it was reported that the best gene editing results was obtained from direct delivery of RNA as opposed to indirect delivery through plasmid DNA [36]. In addition, minimal off-target effects have been observed in direct delivery approach [36, 37].

## Conclusion

In conclusion, we are successful to validate our hypothesis that CRISPR/Cas9-mediated knock- out of *VPREB1* gene in myeloma cells efficiently inhibits their proliferation. This would provide a promising therapeutic target for the management of MM patients in the near future. In addition, large-scale study should be designed to address different pathways that are involved in the proliferation inhibition of myeloma cells by *VPREB1* gene knock-out. This would enhance the therapeutic potential of *VPREB1* gene knock out and highlight also its diagnostic and/or prognostic values in MM and possibly other related hematologic malignancies.

## Supporting information

S1 Fig (PDF)

S1 File (DOCX)

## References

[1] Moreau P, Zamagni E, Mateos M-v. Treatment of patients with multiple myeloma progressing on frontline-therapy with lenalidomide. Blood Cancer Journal. 2019;9(4):1–8. Epub 2019/03/22. 10.1038/s41408-019-0200-1 30894516 PMC6426995

[2] Dimopoulos MA, Ludwig H, Einsele H, Zweegman S, Facon T, Cavo M, et al Multiple myeloma: ESMO Clinical Practice Guidelines for diagnosis, treatment and follow-up † Clinical Practice Guidelines. Ann Oncol. 2017;28(April):52–61. Epub 2017/04/30. 10.1093/annonc/mdx096 . 28453614

[3] Terpos E, Kleber M, Engelhardt M, Zweegman S, Gay F, Kastritis E, et al European myeloma network guidelines for the management of multiple myeloma-related complications. Haematologica. 2015;100(10):1254–66. Epub 2015/10/04. 10.3324/haematol.2014.117176 26432383 PMC4591757

[4] Tian X, Gu T, Patel S, Bode AM, Lee M-h, Dong Z. OPEN CRISPR / Cas9 –An evolving biological tool kit for cancer biology and oncology. npj Precision Oncology. 2019;3(127):8 Epub 2019/03/27. 10.1038/s41698-019-0080-7 30911676 PMC6423228

[5] Sander JD, Joung JKJNb. CRISPR-Cas systems for editing, regulating and targeting genomes. Nat Biotechnol. 2014;32(4):347 Epub 2014/03/04. 10.1038/nbt.2842 24584096 PMC4022601

[6] Cong L, Ran FA, Cox D, Lin S, Barretto R, Habib N, et al Multiplex genome engineering using CRISPR/Cas systems. Science. 2013;339(6121):819–23. Epub 2013/01/05. 10.1126/science.1231143 23287718 PMC3795411

[7] Brouwer JR, Mientjes EJ, Bakker CE, Nieuwenhuizen IM, Severijnen LA, Van der Linde HC, et al RNA-Guided Human Genome Engineering via Cas9 Prashant. Experimental Cell Research. 2013;313(2):244–53. 10.1126/science.1232033 RNA-Guided. 17150213 PMC1852528

[8] Zhan T, Rindtorff N, Betge J, Ebert MP, Boutros M, editors. CRISPR/Cas9 for cancer research and therapy. Seminars in cancer biology; 2019: Elsevier. 10.1016/j.semcancer.2018.04.001 29673923

[9] Zhao C, Zhao Y, Zhang J, Lu J, Chen L, Zhang Y, et al HIT-Cas9: A CRISPR/Cas9 Genome-Editing Device under Tight and Effective Drug Control. Molecular Therapy—Nucleic Acids. 2018;13(December):208–19. Epub 2018/10/13. 10.1016/j.omtn.2018.08.022 30312845 PMC6178243

[10] Lu Y, Xue J, Deng T, Zhou X, Yu K, Huang M, et al A phase I trial of PD-1 deficient engineered T cells with CRISPR/Cas9 in patients with advanced non-small cell lung cancer. Journal of Clinical Oncology. 2018 10.1200/jco.2018.36.15_suppl.3050

[11] Traxler EA, Yao Y, Wang YD, Woodard KJ, Kurita R, Nakamura Y, et al A genome-editing strategy to treat β-hemoglobinopathies that recapitulates a mutation associated with a benign genetic condition. Nature Medicine. 2016;22(9):987–90. Epub 2016/08/16. 10.1038/nm.4170 27525524 PMC5706766

[12] Alateeq S, Ovchinnikov D, Tracey T, Whitworth D, Al-Rubaish A, Al-Ali A, et al Identification of on-target mutagenesis during correction of a beta-thalassemia splice mutation in iPS cells with optimised CRISPR/Cas9-double nickase reveals potential safety concerns. APL Bioeng. 2018;2(4):046103 Epub 2019/05/10. 10.1063/1.5048625 31069325 PMC6481731

[13] Wattanapanitch M, Damkham N, Potirat P, Trakarnsanga K, Janan M, Yaowalak U, et al One-step genetic correction of hemoglobin E/beta-thalassemia patient-derived iPSCs by the CRISPR/Cas9 system. Stem Cell Res Ther. 2018;9(1):46 Epub 2018/02/28. 10.1186/s13287-018-0779-3 29482624 PMC5828150

[14] Skvarova Kramarzova K, Osborn MJ, Webber BR, Defeo AP, McElroy AN, Kim CJ, et al CRISPR/Cas9-mediated correction of the FANCD1 Gene in primary patient cells. International Journal of Molecular Sciences. 2017;18(6):1–15. Epub 2017/06/15. 10.3390/ijms18061269 28613254 PMC5486091

[15] Zhang N, Zhi H, Curtis BR, Rao S, Jobaliya C, Poncz M, et al CRISPR/Cas9-mediated conversion of human platelet alloantigen allotypes. Blood. 2016;127(6):675–80. Epub 2015/12/05. 10.1182/blood-2015-10-675751 26634302 PMC4751021

[16] Park CY, Sung JJ, Cho SR, Kim J, Kim DW. Universal Correction of Blood Coagulation Factor VIII in Patient-Derived Induced Pluripotent Stem Cells Using CRISPR/Cas9. Stem Cell Reports. 2019;12(6):1242–9. Epub 2019/05/21. 10.1016/j.stemcr.2019.04.016 31105049 PMC6565751

[17] Park CY, Kim DH, Son JS, Sung JJ, Lee J, Bae S, et al Functional Correction of Large Factor VIII Gene Chromosomal Inversions in Hemophilia A Patient-Derived iPSCs Using CRISPR-Cas9. Cell Stem Cell. 2015;17(2):213–20. Epub 2015/07/28. 10.1016/j.stem.2015.07.001 . 26212079

[18] Morstadt L, Bohm A, Yüksel D, Kumar K, Stollar BD, Baleja JD. Engineering and characterization of a single chain surrogate light chain variable domain. Protein Science. 2008;17(3):458–65. Epub 2008/02/22. 10.1110/ps.073269808 18287279 PMC2248314

[19] Collins JE, Wright CL, Edwards CA, Davis MP, Grinham JA, Cole CG, et al A genome annotation-driven approach to cloning the human ORFeome. Genome biology. 2004;5(10):R84 Epub 2004/10/06. 10.1186/gb-2004-5-10-r84 15461802 PMC545604

[20] Rossi B, Espeli M, Schiff C, Gauthier L. Clustering of Pre-B Cell Integrins Induces Galectin-1-Dependent Pre-B Cell Receptor Relocalization and Activation. The Journal of Immunology. 2006;177(2):796–803. Epub 2006/07/05. 10.4049/jimmunol.177.2.796 . 16818733

[21] Mårtensson IL, Almqvist N, Grimsholm O, Bernardi AI. The pre-B cell receptor checkpoint. FEBS Letters. 2010;584(12):2572–9. Epub 2010/04/28. 10.1016/j.febslet.2010.04.057 . 20420836

[22] Dc W, Aiba Y, Kameyama M, Yamazaki T, Tedder TF, Kurosaki T. binding to phosphoinositide 3-kinase Regulation of B-cell development by BCAP and CD19 through their binding to phosphoinositide 3-kinase. Blood. 2011;111(3):1497–503. Epub 2007/11/21. 10.1182/blood-2007-08-109769 . 18025150

[23] Winkler TH, Martensson IL. The role of the pre-b cell receptor in b cell development, repertoire selection, and tolerance. Frontiers in Immunology. 2018;9(NOV):1–10. Epub 2018/12/01. 10.3389/fimmu.2018.02423 30498490 PMC6249383

[24] Foundation IM. International Myeloma Working Group (IMWG) Molecular Classification of Multiple Myeloma 2014 [2019-03-01]. Available from: https://www.myeloma.org/classifications-multiple-myeloma.

[25] Millar B, Bell J, Lakhani A, Aylifee M, Selby P, McEtwain TJBjoh. A simple method for culturing myeloma cells from human bone marrow aspirates and peripheral blood in vitro. Br J Haematol. 1988;69(2):197–203. Epub 1988/06/01. 10.1111/j.1365-2141.1988.tb07622.x . 3390392

[26] Ran FA, Nishimasu H, Hsu PD, Konermann S, Shehata S, Dohmae N, et al Double nicking by RNA-guided CRISPR Cas9 for enhanced genome editing specificity. Cell. 2013;154(6):1380–9. Epub 2013/09/03. 10.1016/j.cell.2013.08.021 23992846 PMC3856256

[27] González-Romero E, Martínez-Valiente C, García-Ruiz C, Vázquez-Manrique RP, Cervera J, Sanjuan-Pla AJh. CRISPR to fix bad blood: a new tool in basic and clinical hematology. Haematologica. 2019;104(5):881–93. Epub 2019/03/30. 10.3324/haematol.2018.211359 30923099 PMC6518885

[28] Lucas D, Leary HAO, Ebert BL, Cowan CA, Tremblay CS. Utility of CRISPR / Cas9 systems in hematology research. Experimental Hematology. 2017;54:1–3. Epub 2017/07/03. 10.1016/j.exphem.2017.06.006 . 28668351

[29] Barwick BG, Gupta VA, Vertino PM, Boise LHJFii. Cell of origin and genetic alterations in the pathogenesis of multiple myeloma. Front Immunol. 2019;10:1121 Epub 2019/06/25. 10.3389/fimmu.2019.01121 31231360 PMC6558388

[30] Gundry MC, Dever DP, Yudovich D, Bauer DE, Haas S, Wilkinson AC, et al Technical considerations for the use of CRISPR/Cas9 in hematology research. Exp Hematol. 2017;54:4–11. Epub 2017/08/02. 10.1016/j.exphem.2017.07.006 28757433 PMC5603407

[31] Bejar R, Lord A, Stevenson K, Bar-Natan M, Pérez-Ladaga A, Zaneveld J, et al TET2 mutations predict response to hypomethylating agents in myelodysplastic syndrome patients. Blood. 2014;124(17):2705–12. Epub 2014/09/17. 10.1182/blood-2014-06-582809 25224413 PMC4208285

[32] Liu Z, Chen S, Jin X, Wang Q, Yang K, Li C, et al Genome editing of the HIV co-receptors CCR5 and CXCR4 by CRISPR-Cas9 protects CD4+ T cells from HIV-1 infection. Cell Biosci. 2017;7(1):47 Epub 2017/09/15. 10.1186/s13578-017-0174-2 28904745 PMC5591563

[33] Herzog S, Reth M, Jumaa H. Regulation of B-cell proliferation and differentiation by pre-B-cell receptor signalling. Nature Reviews Immunology. 2009;9(3):195–205. Epub 2009/02/26. 10.1038/nri2491 . 19240758

[34] Kiyokawa N, Sekino T, Matsui T, Takenouchi H, Matsuo Y, Karasuyama H, et al Diagnostic importance of CD179a / b as markers of precursor B-cell lymphoblastic lymphoma. Mod Pathol. 2004;17(4):423–9. Epub 2004/02/21. 10.1038/modpathol.3800079 . 14976526

[35] Chen D, Zheng J, Gerasimcik N, Lagerstedt K, Sjögren H, Abrahamsson J, et al The expression pattern of the Pre-B cell receptor components correlates with cellular stage and clinical outcome in acute lymphoblastic leukemia. PLoS ONE. 2016;11(9):1–16. Epub 2016/09/10. 10.1371/journal.pone.0162638 27611867 PMC5017602

[36] Lino CA, Harper JC, Carney JP, Timlin JA. Delivering crispr: A review of the challenges and approaches. Drug Delivery. 2018;25(1):1234–57. Epub 2018/05/29. 10.1080/10717544.2018.1474964 29801422 PMC6058482

[37] Yu X, Liang X, Xie H, Kumar S, Ravinder N, Potter J, et al Improved delivery of Cas9 protein/gRNA complexes using lipofectamine CRISPRMAX. Biotechnol Lett. 2016;38(6):919–29. Epub 2016/02/20. 10.1007/s10529-016-2064-9 26892225 PMC4853464

