# Supplementary material for: CRISPR/Cas9 mediated knock-out of VPREB1 gene induces a cytotoxic effect in myeloma cells
Source: PLoS One. 2021 Jan 8;16(1):e0245349. doi: 10.1371/journal.pone.0245349 (PMC7794028; doi:10.1371/journal.pone.0245349)
Supplement: S1 Fig — (PDF) [file pone.0245349.s001.pdf]

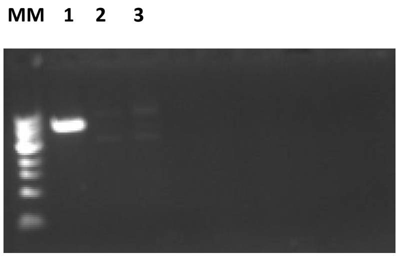

CRISPR/Cas9-mediated cleavage efficiency
Agarose gel image of a cleavage assay using the GeneArt® Genomic Cleavage Detection Assay (Cat. No. A24372) for the CD179a locus. (MW) Molecular weight marker (1 Kb), (1): Results using the GeneArt® CRISPR Nuclease Vector expressing CD179a-specific CRISPR RNA. (2 and 3): Following transfection into primary myeloma cultured cells, cleavage assays were performed.
