# Supplementary material for: CRISPR/Cas9 mediated knock-out of VPREB1 gene induces a cytotoxic effect in myeloma cells
Source: PLoS One. 2021 Jan 8;16(1):e0245349. doi: 10.1371/journal.pone.0245349 (PMC7794028; doi:10.1371/journal.pone.0245349)
Supplement: S1 File — (DOCX) [file pone.0245349.s002.docx]

***+Gene expression and function:*** V-set pre-B cell surrogate light chain 1 (VPREB1): The protein encoded by this gene belongs to the immunoglobulin superfamily and is expressed selectively at the early stages of B cell development, namely, in proB and early preB cells. This gene encodes the iota polypeptide chain that is associated with the Ig-mu chain to form a molecular complex which is expressed on the surface of pre-B cells. The complex is thought to regulate Ig gene rearrangements in the early steps of B-cell differentiation. Alternative splicing results in multiple transcript variants.

***Bioinformatics analysis:***

An in silico as well as *in vitro* analyses were conducted according to the following schedule:

**Bioinformatics Analysis for Biomarker filtration and Target Determination**

The Multiple Myeloma associated genes were filtered from bioinformatics databases such as DisGeNET “<http://www.disgenet.org/search>”, and the Human Gene Mutation Database “<http://www.hgmd.cf.ac.uk/ac/index.php> “, then gene expression profile was downloaded from the Gene Expression Omnibus database.

In the present study, a biological bioinformatics approach was applied in order to analyze the gene expression profiles in Multiple Myeloma patients, a differential expressed genes (DEGs) functional analysis was performed between MM patients and healthy control group. In addition, in order to analyze the DEGs at functional level, we performed a gene ontology (GO) “https://www.uniprot.org/help/gene_ontology

”, Kyoto Encyclopedia of Genes and Genomes (KEGG) “[https://www.genome.jp/kegg” and REACTOME pathway enriched analyses” https://reactome.org”](https://www.genome.jp/kegg) using the Database for Annotation, Visualization and Integrated Discovery (DAVID) online tool “https://david.ncifcrf.gov/” , to determine the molecular functions (MFs) and biological pathways. A p-value was set as the threshold value. A screen shots were taken for the selected gene “V-set pre B-cell surrogate light chain 1”VPREB1” as shown in figure 1, 2.

**Figure 1.** A screen shot representing the bioinformatics analysis that was conducted on VPREB1 gene expression in Multiple Myeloma using *DisGeNET* database

C

**Figure 2.** A screen shot representing the bioinformatics analysis that represents the evidence of Multiple Myeloma – VPREB1gene association using *DisGeNET* database

**The used Gene editing assay:**

we used a commercial gene editing assay “True crRNA assay for VPREB1 gene editing [ID CRISPR812534_CR] (***Invirogen, ThermoFisher, Germany***), the selection of the assay was based on the highest score recorded for VPREB1 gene editing which reaches 93.18%. Figure 3 and 4 illustrates a screen shots for the crRNA and primer sequence of the assay. In the manuscript, we were stratified with presenting the assay cat no, ID and the company name, otherwise, these data are available on the Thermofisher Scientific website. Herein, the fully data regarding the full data of the used assay.

**Figure 3.** A screen shot representing the selection of Cas_9 associated crRNA assay for knock out of VPREB1gene using *ThermoFisher gene editing* software

**Figure 4.** A screen shot representing the criteria and sequence of the of Cas_9 associated crRNA assay for knock out of VPREB1gene using.
